# Application of a New Geophone and Geometry in Tunnel Seismic Detection

**DOI:** 10.3390/s19051246

**Published:** 2019-03-12

**Authors:** Yao Wang, Nengyi Fu, Xinglin Lu, Zhihong Fu

**Affiliations:** 1 State Key Laboratory of Power Transmission Equipment and System Security and New Technology, Chongqing University, No. 174 Shazhengjie, Chongqing 400044, China; 20161101003@cqu.edu.cn (Y.W.); xinglinlu1992@163.com (X.L.); 2 School of Electrical Engineering, Chongqing University, No. 174 Shazhengjie, Chongqing 400044, China; 3 Depatrment of Geophysics, Colorado School of Mines, Golden, CO 80401, USA; nengyifu@mymail.mines.edu

**Keywords:** tunnel seismic, semi-automatic coupling geophone, geometry, horizontal offset, vertical offset

## Abstract

Seismic imaging is the most effective geophysical method and has been extensively implemented to detect potential geological hazards in tunnels during construction. The coupling of geophones and the design of geometry in tunnels are the two major challenges. To ensure successful coupling, a high-sensitivity semi-automatic coupling geophone with a broadband was designed. In practice, this geophone is attached with a wheel and two springs. Once inserted into the borehole, an automatic coupling action occurs. This semi-automatic coupling design within the geophone not only guarantees good coupling, but reduces the time and costs usually required to install a traditional geophone. In the use of geophones for tunnel seismic detection, we propose two new two-dimensional (2D) seismic geometries based on the two commonly used geometries. A test to assess the effectiveness of the qualities of imaging from four geometries was completed by comparing the results of the forward modeling of sandwich models. The conclusion is that the larger the horizontal offset of the layout geometry, the higher the resolution of the imaging; the larger the vertical offset, the weaker the mirror image. The vertical offset is limited due to the narrow tunnel condition. Therefore, the mirror effect cannot be entirely eliminated; however, it can be further suppressed by constructing 2D geometry. The two newly proposed 2D geometries caused the imaging arc of the inter-layer, but suppressed the mirror image. The mirror image added a significant number of errors to the data, which could misguide tunnel construction; therefore the new 2D geometries are more reasonable than the two most commonly used. We applied one of the two new 2D geometries that was more practical to an actual project, the Chongqing Jinyunshan Tunnel in China, and acquired high-quality seismic data using two semi-automatic coupling geophones. The detection results were essentially consistent with the excavation conclusions.

## 1. Introduction

Infrastructure and energy projects, such as transportation, water resource transportation, municipal pipelines, and mining, all involve tunnel construction. As society develops, increasingly extensive use of underground and natural resources is occurring, so safety during the tunnel construction stage is a major issue requiring immediate attention [1,2]. Due to the limitations of the previous subsurface geological exploration, accurately imaging all details, such as small faults, weak layers, karst caves, and goafs in front of the tunnel face, is difficult, especially for the construction of long tunnels [3,4]. Seismic imaging is one of the optimal methods for cases like this due to its exclusive advantages of in-depth detection and high resolution [5,6,7]. 

The theory of tunnel seismology is mainly based on oil seismology. The processing flow resembles the Vertical Seismic Profile (VSP) method [8,9]; however, the coupling of the geophone differs from oil seismology to some degree. In oil seismology, the tails of the geophone are buried vertically into the surface soil to achieve better coupling [10]. As a tunnel essentially consists of hard rocks, the coupling is difficult. At present, three main coupling methods are used in the industry. The first method is the casting method [7]. To perform this method, first, a 2-m-deep hole is drilled into the tunnel wall and an anchoring agent is pushed into the hole with a push rod before a special-made steel pipe is pushed into the borehole. The length of the steel pipe is 2 m and the cross-section is a square with a side length of about 3 cm. Second, the steel pipe is struck with a heavy hammer to fully insert the front end of the steel pipe into the anchoring agent inside the drill hole, before waiting for the anchoring agent to solidify. At this time, the steel pipe is firmly bonded into the tunnel wall, and the geophone is pushed with a special-made push rod. The steel pipes are designed to fit geophones, so the size of the geophone is slightly smaller than the size of the steel pipes. Taking geophones out for reuse is convenient for workers with this user-friendly design. This method significantly reduces acoustic and surface waves and the influence of low speed circle that are caused by excavation. This method has the following shortcomings. First, as the geophone can be pushed in and out freely in the steel pipe, the coupling is not sufficiently tight; second, this method is time-consuming and requires about half an hour to install one geophone; finally, it is expensive. Once the steel pipe has been tightly bonded to the tunnel wall, it cannot be taken out for reuse. The cost of each data acquisition in steel pipes and anchoring agents is about $700 dollars. The second method is the clay coupling method, which is widely used in China [11]. With this method, a 2-m-deep hole in the tunnel wall is first drilled and clay is placed into the borehole before the geophone is pushed into the clay. The clay fills the gaps between the geophone and borehole to ensure good coupling. This method is cheaper, but is unable to address the other two issues of the casting method. The third method is the direct coupling method, wherein the geophone is directly attached to the tunnel wall with quick-drying cement [12]. However, this method acquires a large amount of noise, such as acoustic waves and surface waves, and also fails to avoid the influence of the tunnel surface low speed circle caused by excavation. To address these coupling issues, we designed a wide-band and high-sensitivity semi-automatic coupling geophone.

An ideal seismic survey, consisting of a dense grid of shots and geophones, can produce and record the ideal seismic wavefield perfectly. Seismic geometry is the space coordinates of the shots and geophones. In oil seismic exploration, sparse geometry (such as a parallel, orthogonal, or other irregular geometry) is often adopted in practical seismic exploration applications according to different exploration targets [13]. However, the narrow space of the tunnel limits the length of the offset (the distance between the shot and the geophone). Designing viable small offset geometry that can accurately detect unexcavated regions remain a challenging task in the industry. Dickman and Sander proposed the Tunnel Seismic Prediction (TSP) method, in which the geometry is arranged on the side wall of the tunnel, 24 shots are set along one line on one tunnel side, and two geophones are arranged on the same and opposite sides of the shots [14]. Inazaki et al. proposed the Horizontal Sonic Profiles (HSP) method, which was intended for Tunnel Boring Machine (TBM) tunneling. The geometry requires fewer shots but more geophones, and places the geophones on the same level in the tunnel [15]. Neil et al. proposed the True Reflection Tomography (TRT) method, which adopts the geometry of spatial multi-point excitation and multi-point reception. Geophones and source points are arranged on both sides of the tunnel and the top plate. However, this geometry is only suitable for hammer seismic sources [16]. Kneib et al. proposed the Sonic Soft ground Probing (SSP) system, which uses ultrasonic sources instead of explosives. The sources and geophones are mounted on the TBM cutterhead, and the cutterhead is placed close to the tunnel face to acquire the data [17]. Borm et al. proposed the Integrated Seismic Imaging System (ISIS) system, in which the geophone is attached to the tunnel anchor and a hammer or other artificial source is used to excite the seismic wave at any position on the tunnel face to form a geometry [18]. The above geometries have their own characteristics, and successful detection data have been obtained in different tunnels. However, few studies have been published on the design principle of geometry. In this study, two two-dimensional (2D) geometries suitable for tunnels are proposed. Based on the typical weak inter-layer model, the geometry and the conventional linear geometry were forward modeled. The influence of the vertical offset and the horizontal (tunneling direction) offset on the imaging effect was analyzed. Finally, we applied the semi-auto coupling geophone and geometry in the Jinyunshan Tunnel, China.

## 2. Semi-Automatic Coupling Geophone

### 2.1. Sensors

**Figure 1:**
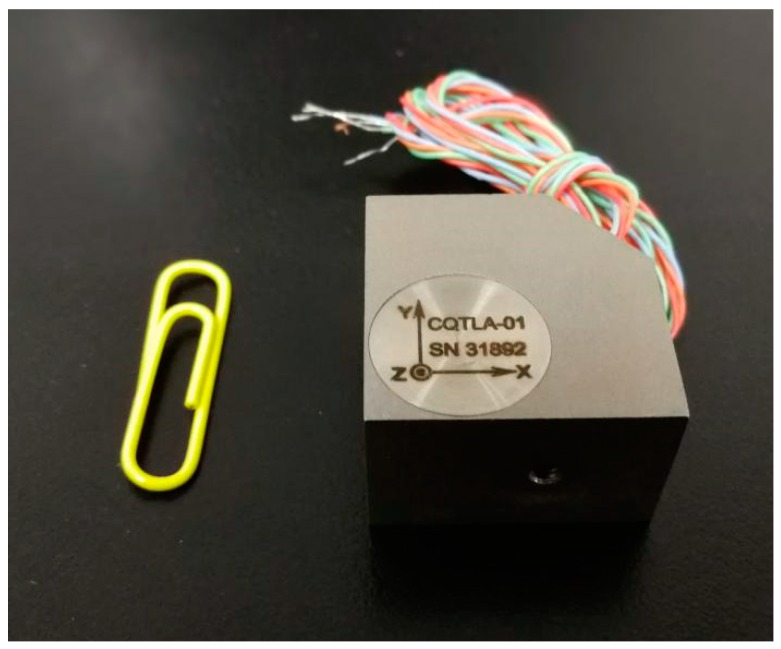
The three-component piezoelectric sensor.

**Figure 2:**
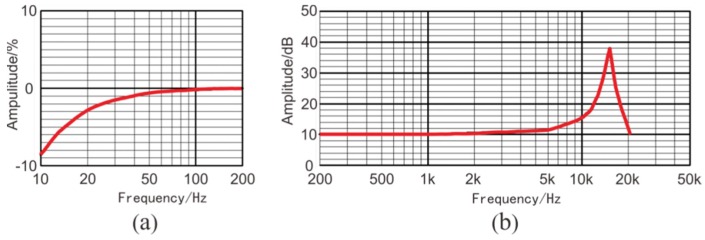
The amplitude responses of the three-component piezoelectric sensor: (**a**) low-frequency, (**b**) medium- and high-frequency.

Two main types of sensors are commonly used in seismic geophones: the coil-based sensor and the piezoelectric sensor [19,20]. Coil-based sensors are rugged, cheap, and self-powered [21]; however, they have a narrow bandwidth spectrum and low sensitivity. In tunnel seismic exploration, the offset is normally small, so the signal spectrum can be up to 2 KHz. Coil-based sensors do not meet the requirements of tunnel seismic exploration. Tunnel seismic data include strong amplitudes of direct waves, acoustic waves, surface waves, and tunnel face reflections, whereas the reflected waves from the geological body of the advanced tunnel face are relatively weak. Coil-based sensors are unsuitable for capturing weak signals like these; therefore, a three-component piezoelectric sensor was selected (Figure 1) to ensure that the geophone had a spectrum range of 10 to 5000 Hz and a sensitivity of 2.8 V/g. These sensor parameters satisfy the requirements for tunnel seismic detection. The specifications of the three-component piezoelectric sensor are shown in Table 1, where x y and z represent the X-component, the Y-component, and the Z-component, respectively. The amplitude responses are shown in Figure 2. The sensor was tested using two vibration platforms: Figure 2a shows the low-frequency signals and Figure 2b shows the medium- and high-frequency signals.

### 2.2. Semi-Automatic Coupling Geophone

**Figure 3:**
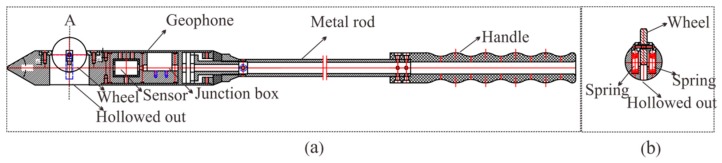
Cutaway view of the semi-automatic coupling geophone: (**a**) a side view, (**b**) a cross-section view of wheel.

**Figure 4:**
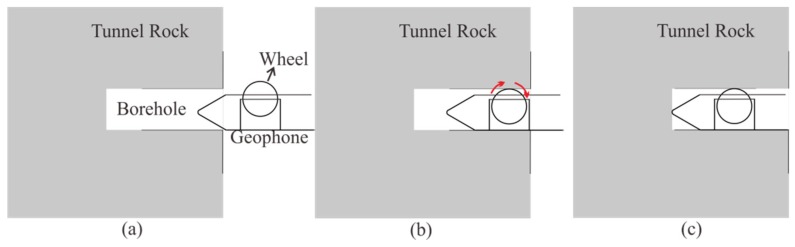
The semi-automatic coupling geophone installation process: (**a**) before, (**b**) during, and (**c**) after installation.

To guarantee the high quality of the seismic data, our method still requires drilling a 2-m-deep hole to install the geophones. The design of the semi-automatic coupling geophone is shown in Figure 3. Figure 3a presents a side view where the device consists of three parts: the geophone, a metal rod, and a handle, from left to right. The geophone is composed of a piezoelectric sensor, a wheel, and a junction box. The wheel is used for coupling. Figure 3b provides a cross-section view of wheel. The center of the wheel is connected with two hard springs so that the wheels can rotate and move in the vertical direction. Once the device has been pushed into the borehole, the wheel starts to roll along the surface of the sidewall of the borehole, which facilitates the installation of the geophone. In addition, its tight coupling and easy removal are ensured. The process of installing the semi-automatic coupling geophone is shown in Figure 4. After the whole device has been pushed into the borehole, a wedge-shaped stone block is applied to clamp the tail of the device to prevent the vibration of the tail at the end of the rod. Afterward, the hole is sealed with clay to reduce the sound waves. However, the rolling approach creates new problems as considerable amounts of rock slag remain in the tunnel borehole and the rolling process will force the slag into the cavity below the wheel, which will eventually block the wheel. Therefore, the sleeve under the wheel was designed to be hollowed out, as shown in Figure 2. The wheel was directly washed after each use, so that the slag could be directly discharged from the hollow cavity below the wheel. 

**Figure 5:**
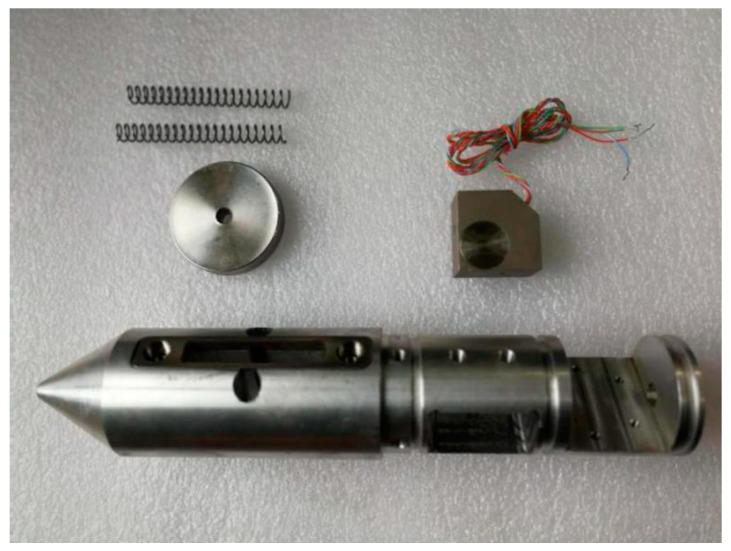
Internal structure diagram of the semi-automatic coupling geophone.

**Figure 6:**
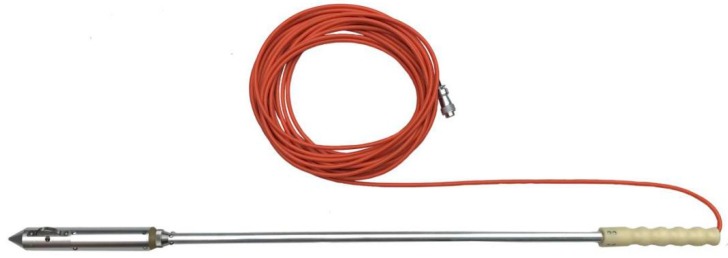
Picture of a semi-automatic coupling geophone.

Figure 5 and Figure 6 show the internal structure diagram and a picture of a semi-automatic coupling geophone, respectively.

## 3. Tunnel Seismic Geometry

### 3.1. Geological Model

**Figure 7:**
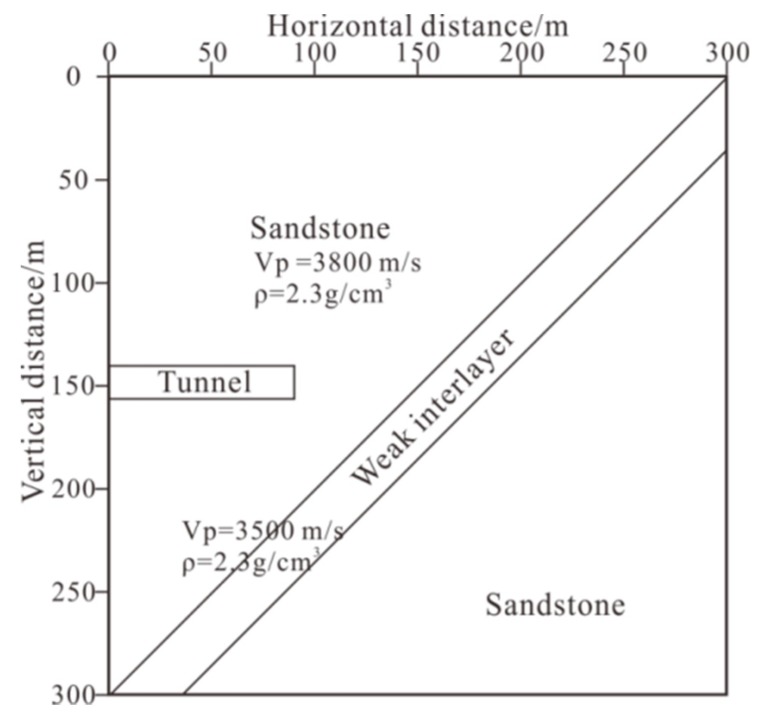
Sketch of the geological model.

The geological model was established as shown in Figure 7. The model was a square with a side length of 300 m. 

The model mainly consisted of sandstone, but with a sloping weak inter-layer in the middle. The weak inter-layer had a length of 60 m in front of the tunnel face and the thickness was 25 m. In Figure 7, V_p_ is the primary wave velocity and ρ is the density.

### 3.2. Tunnel Seismic Geometry

**Figure 8:**
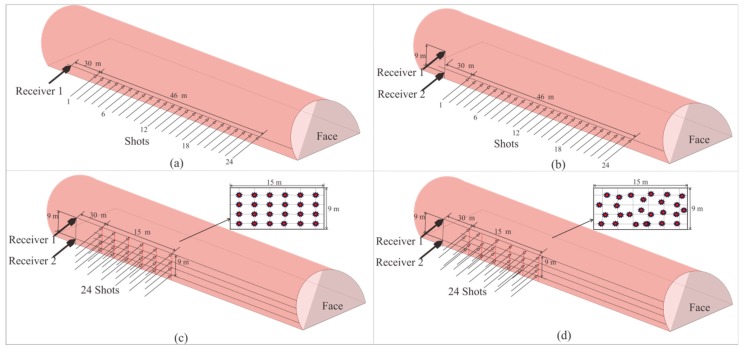
The four geometries used in this study, where the minimum offset was 30 m, the borehole span was 2 m, and the spacing for the vertical geophones was 9 m: (**a**) 24 shots and 1 geophone, shots were arranged along a straight line; (**b**) 2 geophones were placed at the top and bottom of the tunnel wall. The vertical location of shot line was 4.5 m. Compared with the first geometry, the second geometry had an additional geophone and vertical offset. (**c**) The shots were arranged in a 2D 15 m × 9 m plane. The red pots are the shot locations. The locations of two geophones were the same as the second geometry. Compared with the second geometry, this geometry has a shorter horizontal offset and more vertical offset. (**d**) The ventilating pipe, water pipe, and machines obstruct the desired layout of the third grid geometry in the environment of the tunnel construction. Thus, the 24 shots were randomly positioned in the 2D plane. Compared with the third regular geometry, the fourth geometry design layout is more likely to be used in complex tunnel environments.

In this study, forward models of four different tunnel seismic geometries were applied (Figure 8) based on same velocity model (Figure 7). The first two geometries were derived from the TSP method, which were slightly improved for the model. The third geometry was the new one proposed in this paper. This geometry is more 2D, thus can provide more vertical offset data. The fourth geometry was an improvement based on the third geometry. Due to the poor condition of the tunnel construction, the geophones were not expected to be arranged in the desired rectangular grid manner.

### 3.3. Numerical Calculations

Based on the acoustic wave equation, we numerically simulated the above geometry. The two-dimensional wave equation in an acoustic, isotropic, and heterogeneous medium is described as follows:

(1) $$\frac{1}{v_{p}{}^{2}}\frac{\partial^{2}u}{\partial^{2}t}=\frac{\partial^{2}u}{\partial x^{2}}+\frac{\partial^{2}u}{\partial z^{2}}$$

where u = u(x,z,t) is a scalar wave field, and v_p_ = v_p_(x,z) is the velocity field. Using the Taylor series expansion, the time derivative is a second-order center difference, and the spatial derivative is a 2N-order difference.

(2) $$\begin{array}{l} u_{i,j}^{k+1}=2u_{i,j}^{k}-u_{i,j}^{k-1}+\frac{1}{2}{\left(\frac{v_{p}\Delta t}{\Delta x}\right)}^{2}\left[a_{0}u_{i,j}^{k}+\overset{N}{\underset{n=1}{\sum }}a_{n}\left(u_{i+n,j}^{k}+u_{i-n,j}^{k}\right)\right]+\\ \frac{1}{2}{\left(\frac{v_{p}\Delta t}{\Delta z}\right)}^{2}\left[a_{0}u_{i,j}^{k}+\overset{N}{\underset{n=1}{\sum }}a_{n}\left(u_{i,j+n}^{k}+u_{i,j-n}^{k}\right)\right] \end{array}$$

where Δx and Δz are the space step, Δt is the time step, and a_n_ is the difference weight coefficient:

(3) $$a_{n}=\frac{{\left(-1\right)}^{n+1}\prod_{i=1,i\neq n}^{N}i^{2}}{2n\prod_{i=1}^{n-1}\left(n^{2}-i^{2}\right)\prod_{i=1+n}^{N}\left(i^{2}-n^{2}\right)},n=1,\ldots ,N$$

When $\Delta t\leq \frac{\Delta x}{v{\sqrt{2}}_{max}}$is satisfied, the solution is stable. The space step of the model was 0.7 m. A 200 Hz Ricker wavelet was used as the source, and the boundary conditions were selected as the Perfectly Matched Layer (PML) boundaries, and the above conditions were used for the programming calculation.

### 3.4. Tunnel Seismic Geometry Forward Modeling Result

**Figure 9:**
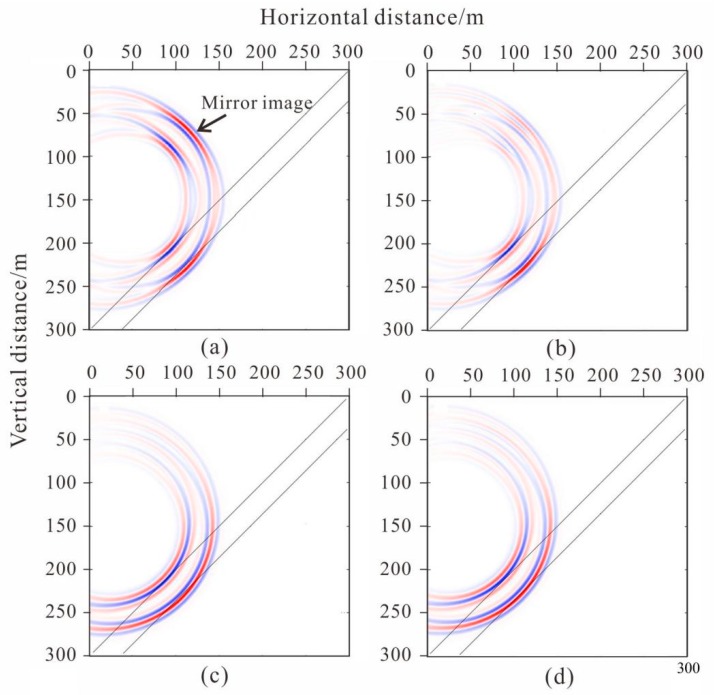
Migration profile of four geometries. (**a**), (**b**), (**c**) and (**d**) correspond to Figure 8a–d, respectively.

Figure 9 illustrates the imaging results obtained by the pre-stack Kirchhoff migration after removing the direct wave of the forward seismic data. For comparison, the results from the four geometries are presented. 

The geometry could clearly image the weak inter-layer; however, mirror imaging occurred in the migration profile (Figure 9a) and the amplitude of the mirror imaging was the same as the image of the weak inter-layer. Compared with the migration imaging of the first tunnel seismic geometry (Figure 9a), the mirror imaging of the second tunnel seismic geometry was diminished (Figure 9b). The mirror imaging of the third tunnel seismic geometry was weaker than in the second, although this migration imaging obviously prolonged the length arc in the third geometry than in the previous two (Figure 9c). The migration profiles of the fourth geometry were almost the same as the third geometry (Figure 9d). The mirror imaging of the last two geometries were greatly suppressed, although the arc was obvious. In the construction of tunnel engineering, the harm caused by mirror imaging is much greater than that of the arc because the arc only reduces the resolution, whereas the mirror image is wrong and not the true reflection of the geological body. Therefore, the geometries we propose are more reasonable and can be used in practical applications.

## 4. Engineering Applications

### 4.1. Geological Conditions

The Jinyunshan Tunnel is a long inter-city tunnel that circles around Chongqing, China in the parallel ridge valley area in the eastern part of the Sichuan Basin. Along the tunnel, the outcrops are as follows: Quaternary clay, silty clay and massive sandstone, the lower Shaximiao~Zhuchong formation of Jurassic, and the Xujiahe~Jialingjiang formation of Triassic period. The tunnel passes through six sections of coal-bearing strata. There are 29 coal mines of different sizes that have been mined at different periods on the north and south sides of the tunnel. The mining operations were shut down in the 1930s and 1940s. At present, there are no related coal mine data, tunnel seismic detection is necessary to guide the safe construction of the tunnel.

### 4.2. Seismic Data Acquisition

The geometry in Figure 8d was chosen for our tunnel seismic survey, with 24 shots and two geophones. The data were obtained via a TETSP-2 tunnel seismograph (Chongqing Triloop Detection Co., Chongqing, China). The basic parameters of the TETSP-2 tunnel seismograph are shown in Table 2. 

**Figure 10:**
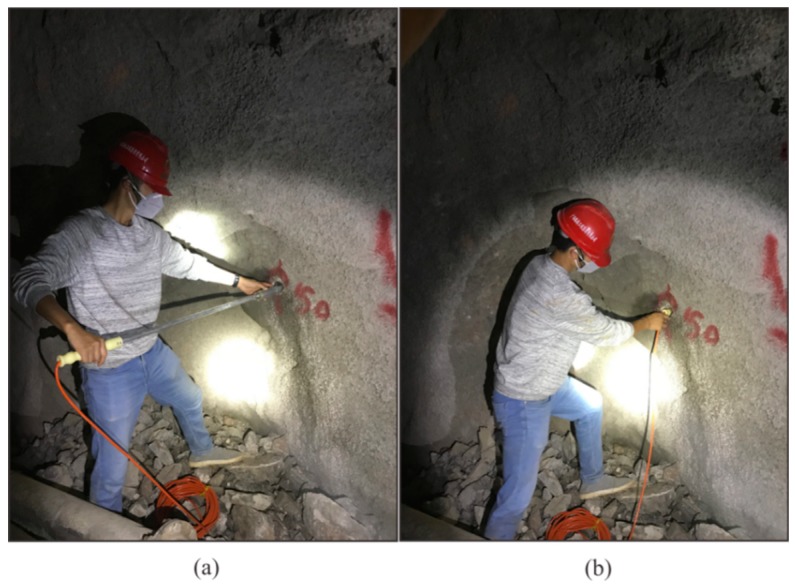
The semi-automatic coupling geophone installation in the tunnel: (**a**) before, (**b**) after installation.

Figure 10a,b show the semi-automatic coupling geophone installation process. The entire process was completed in less than one minute and does not require any coupling tool, so there are no material costs.

### 4.3. Seismic Data Processing and Interpretation

**Figure 11:**
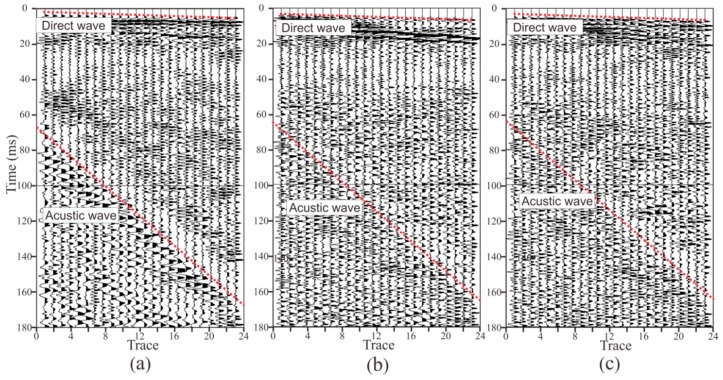
Seismic data acquired by geophone 1. There are three components of data, x, y, and z, where the x direction is the tunnel excavation direction, the y direction is the direction of the borehole where the geophone is mounted, and the z direction is the vertical direction: (**a**) x-component, (**b**) y-component, (**c**) z-component.

**Figure 12:**
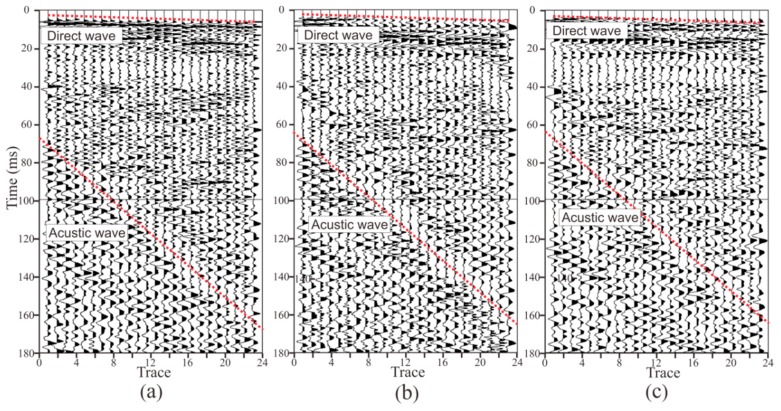
Seismic data acquired by geophone 2. The three components of data are x, y, and z, where the x direction is the tunnel excavation direction, the y direction is the direction of the borehole where the geophone is mounted, and the z direction is the vertical direction: (**a**) x-component, (**b**) y-component, (**c**) z-component.

Figure 11 and Figure 12 present the seismic data obtained by two semi-automatic coupling geophones. The x-, y-, and z-component data are similar, indicating that the consistency of the three-component sensors was good. The seismic signal in the tunnel is more complicated than the oil seismic signal. As the oil seismic wave propagates in half space, and the velocity of the medium is faster from shallow to deep, the seismic wave is continuously refracted during the propagation. When the depth of detection is several kilometers, the seismic wave is assumed to be normally incident and reflected, and the propagation path of the seismic signal is relatively simple. In tunnel seismology, since the detection distance is within 200 m, the seismic wave is a spherical wave that propagates in the whole space. Therefore, the seismic wave propagation path is complicated and can be transmitted from almost any direction of the whole space. The seismic signals in Figure 11 and Figure 12 mainly include direct waves, surface waves, the reflected wave of the face, acoustic waves, the reflected wave of the geological body in all directions of the tunnel, and various noise signals in the tunnel. These seismic waves overlap most of the others, so only the direct wave and the acoustic waves (red dashed lines) can be found in Figure 11 and Figure 12. The upper red dotted line in Figure 11 and Figure 12 represents the direct wave. Converting the abscissa in the figure into the offset, the reciprocal of the slope of the line is the velocity of the direct wave. The velocity of the direct wave is an important parameter for understanding the geological conditions of the rock, and is also an important parameter for subsequent data processing. The direct wave was clear and presented as a distinct line, indicating that the quality of the acquired data was high. The red dotted line in the lower part of Figure 11 and Figure 12 is the acoustic wave. As the explosive was excited in the rock and the tunnel space was narrow, the acoustic wave energy was strong, so it was be clearly identified, as shown in Figure 11 and Figure 12. Converting the abscissa in the figure into the offset, the reciprocal of the slope of the line was the velocity of the acoustic wave. The sound wave was removed as a noise signal during processing. However, since the velocity of the acoustic wave was stable, the data could be used to verify whether the measurement of the field geometry was accurate.

**Figure 13:**
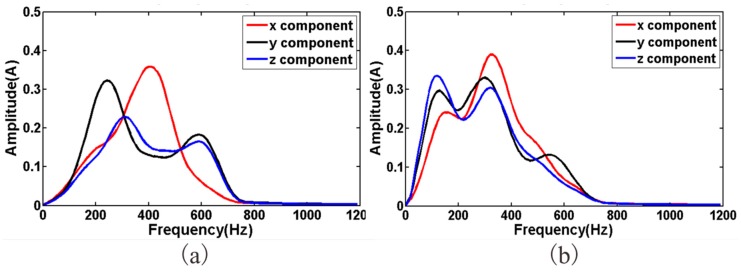
Spectral analysis of the seismic data acquired by two geophones: (**a**) spectrum of Figure 7, (**b**) spectrum of Figure 8.

The spectrum of the seismic data is shown in Figure 13. The spectrum of the three components was similar, indicating that the consistency of three-component sensors was good. The spectral range of the data acquired by the two geophones was approximately 10–700 Hz and 10–650 Hz, whereas the spectrum range of the conventional oil seismic signal is generally 10–60 Hz. From the perspective of signal analysis, the wider the spectrum range, the higher the resolution, which means that these semi-auto coupling geophones acquired wide-band tunnel seismic signals that can be supported by subsequent high-resolution processing.

**Figure 14:**
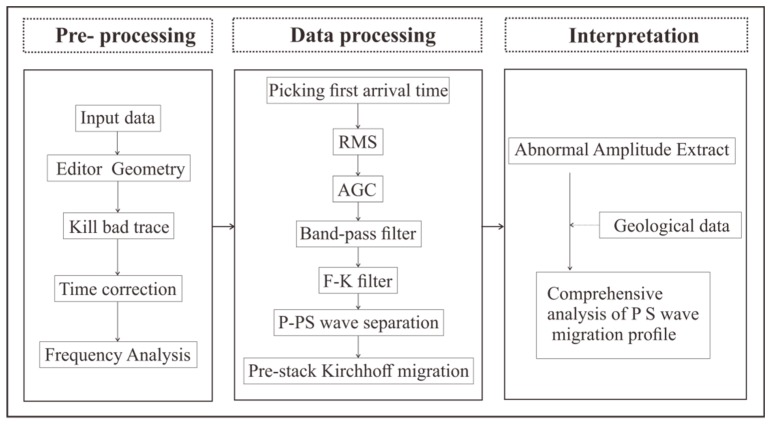
Seismic data processing flow chart.

The purpose of tunnel seismic processing is to extract the reflected waves of the geological body in front of the tunnel face from the complex signals and then image them. The data processing flow is illustrated in Figure 14, including the three major steps: pre-processing, processing, and interpretation. The purpose of pre-processing is to input data and prepare it for subsequent processing. First, the seismic data are input, then the geometry is edited, and then the bad trace of seismic data is eliminated. In a few cases, the instrument triggers a delay, and the time correction can be used to correct the data. Spectrum analysis is used to display the spectrum of the raw data. Processing is mainly used to extract and image the effective wave-field. We selected first arrival time of seismic wave to calculate the direct wave velocity. The Root Mean Square (RMS) is used to equalize the amplitude of the seismic profile. Since the seismic wave is a spherical wave, the energy decays exponentially with the propagation time. Therefore, the Automatic Gain Control (AGC) is used to recover the bottom data amplitude. The band-pass filter is used to filter out the surface waves, acoustic waves, and some other noise signals in the tunnel. One fundamental difference between tunnel seismic data processing and conventional oil seismic data processing is that seismic waves propagate in full space in the tunnel seismic, whereas they propagate in half space for oil seismic waves. This leads to a significant increase in the difficulty in data processing. The F-K (where F is frequency, K is wave-number) filter can be applied to transform the seismic signal from the time domain to the frequency wave-number domain to extract the reflected signal from the advanced tunnel face [22]. The P- and S-waves are separated from the three-component VSP data via the wave separation method [23]. The three-component seismic data is further separated into P, Sh, and Sv waves. The pre-stack Kirchhoff migration is applied after separation to image the P, Sh, and Sv waves. The tunnel seismic interpretation method is also different from that of the conventional oil seismic method. The tunnel seismic detects the medium in front of the face, so the direction of the seismic waves’ propagation is parallel to the layered medium rather than perpendicular to the layered medium. Therefore, identifying distinctive layer boundaries is difficult, even in the migration profile. Extraction from the anomalous amplitude on the migration profile is recommended to interpret the data. Normally, a combination of the geological data and P wave, Sh wave, and Sv wave migration profiles is needed to analyze the geological conditions in front of the tunnel face.

**Figure 15:**
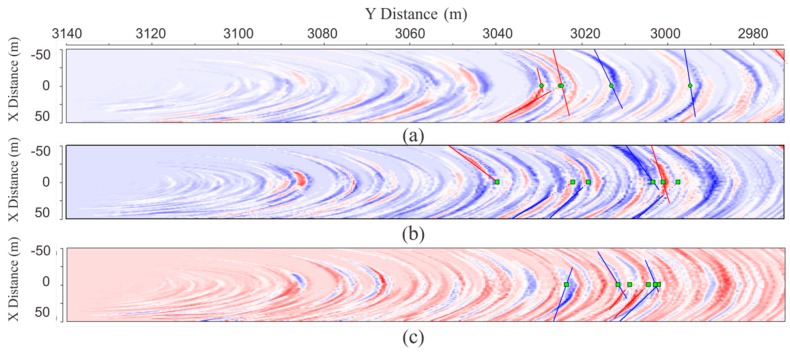
Seismic migration profile where X is the tunnel excavation direction and Y is the vertical direction: (**a**) P wave, (**b**) Sh wave, and (**c**) Sv wave.

**Figure 16:**
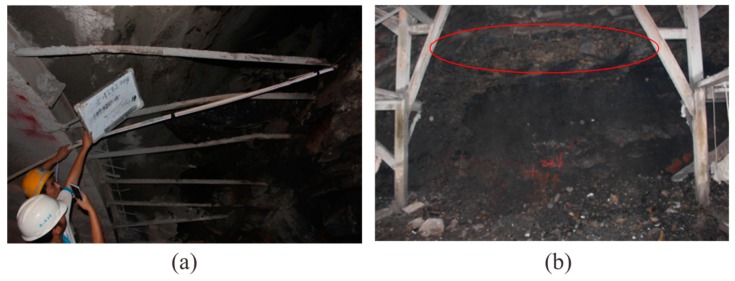
Tunnel face at (**a**) K3 + 024.5 and (**b**) K3 + 021.

The seismic migration profile is shown in Figure 15. Through the amplitude anomaly attribute extraction from Figure 15, the P-wave and both the Sh and Sv waves encountered several large impedance differences between the mileage at K3 + 030 and K3 + 005, where a few of the strongest magnitudes of colors existed. Combined with the geological conditions, we speculated that the region is relatively heterogeneous and fragmented, with possible goafs. Our conclusion for the excavation is as follows: an old tunnel for mining was exposed at K3 + 024.5 (Figure 16a). The old tunnel almost traversed the face of the new tunnel, and the mileage difference from the intersection of the left and right side of the walls of the new tunnel was about 1 m. The width and height of the old tunnel were about 1.2 m and 1.5 m, respectively. The bottom of the old tunnel was almost parallel to the new tunnel floor, which was 6 m higher than the new tunnel. The bottom medium of the old tunnel was alternately composed of layers of thin coal and rock layers. At K3 + 021, there was a goaf (red line in Figure 16b) located 1.7 m above the tunnel floor with a layer thickness of 1.2 m. This was backfilled with vermiculite and had a breccia-like loose structure with extremely poor stability. K3 + 019–018 was mainly composed of shale, with a coal seam and vermiculite backfill. The vault part of this section was extremely broken. The K3 + 018–009 section was a strongly weathered carbonaceous shale with a loose structure that was extremely broken with poor stability. The homogeneity and integrity of the surrounding rock gradually became better after K3 + 006.

## 5. Discussion and Conclusions

An innovative semi-automatic coupling geophone was designed in this study to tackle existing issues in tunnel seismic detection. This device was equipped with a piezoelectric sensor with a spectral width of 10–5,000 Hz and a sensitivity of 2.8 V/g, which greatly exceeds the traditional coil based geophone. The standard requirements for tunnel seismic signal acquisition can be met using this device. A wheel and springs were designed to ensure that the geophone could be well coupled to the hole wall. Compared with the previous method, the method guarantees a more direct contact between the geophone and the tunnel wall without using a casing or coupling agent, which ensures that high quality raw data are obtained. The installation time was reduced from 30 minutes to 1 minute when compared to a traditional geophone, which greatly contributes to the improvement in its efficiency. Work efficiency is one of the most essential aspects in tunnel seismic data acquisition because tunnel seismic detection is performed during the tunnel construction stage, when the environment in the tunnel is noxious. Dust and a lack of air can be harmful to worker health. Longer working times may affect the connection between the various processes in the tunnel construction. The semi-automatic coupling geophone is also economical and does not require a casing and coupling agent.

Forward modeling studies were completed on four tunnel seismic geometries based on a sandwich model. Since the model was relatively simple and the migration velocity directly used the model velocity, the inter-layer was accurately imaged. However, a mirror image still appeared in the migration profile of the first geometry. This is because the first geometry is a one-dimensional array that cannot determine whether the reflected waves propagated from above or below. The mirror image of the second geometry was obviously weakened in the migration profile because the amount of data was doubled by adding a geophone. More importantly, the additional geophone caused the two-dimensional geometry and the vertical offset to appear. Kirchhoff migration is an imaging method that uses the different incident paths of the data weighted superposition. The vertical offset created more ray path data, so the real reflection surface was strengthened and the mirror image was relatively suppressed as a result. We can understand the mirror image from such a perspective because the seismic wave is a spherical wave rather than a plane wave, so the mirror image was obvious in the first geometry. The second geometry had some vertical offset. Under this condition, the seismic wave was a bit like a plane wave, so the image was weaker. If the vertical offset is several times larger than the vertical width of the inter-layer, the seismic wave can be thought of as a plane wave, and the mirror can be completely eliminated. However, under actual conditions, the vertical width of the tunnel is often smaller than the geological body in the direction ahead of the tunnel face. The mirror image in the migration profile of the third geometry was weaker than that of the second geometry. This geometry had the same number of shots and geophones as the second, but its shot distribution was more two-dimensional and the angle of the ray was more diverse. The inter-layer imaging showed an obvious arc, which indicated that the resolution was lower than the migration profile of the second geometry. This occurred because the horizontal offset of the geometry was shorter than that of the previous one. For the inter-layer, large offset data in the horizontal direction were missing, so the resolution was lower. The migration profiles of the fourth geometry were almost the same as the third geometry, and this kind of geometry is more realistic for practical applications. From this, we infer that the larger the horizontal offset of the geometry, the higher the resolution of the imaging; the larger the vertical offset, the weaker the mirror image. As the limited vertical offset was due to the height of the tunnel, completely eliminating the mirror was difficult, but could be further suppressed by constructing a two-dimensional geometry. Although previous studies have proposed more complex geometry, such as the ISIS, HSP, and TRT methods, none of these methods have analyzed the effect of vertical and horizontal offset on imaging and the principle of the further suppression of the mirror image. The third and fourth geometries enriched the vertical offset data, although this was at the cost of reducing the horizontal offset data. This design reduced the resolution, but suppressed the mirror image. The mirror image often leads to a misguided tunnel construction, so two-dimensional geometry is more suitable for investigations like these.

The fourth geometry was applied to the Jinyunshan Tunnel in Chongqing, China where we designed the tunnel seismic data processing and interpretation process. In the anomalous regions of the migration profile, the surrounding rock was relatively broken, and an old tunnel and goaf were excavated. This indicated that the geophone and the geometry were feasible and could provide a safety reference for tunnel construction.

The design for three-dimensional seismic detection in tunnels will be the subject of our future interest. Based on the two-dimensional geometry proposed in this paper, the three-dimensional seismic detection of tunnels can be realized by adding geophones to the opposite side walls of the tunnels. By fully using the horizontal direction (tunnel excavation direction) length and rationally using the limited distances of the other two directions, three-dimensional imaging of the tunnel face can be realized. In addition, three-dimensional seismic detection requires more geophones. The traditional installation of geophones is time-consuming and costly. Semi-automatic coupling can greatly improve the working efficiency and support the seismic detection of 3D tunnels. This study also has the following limitations. The two-dimensional model has certain limitations, and can not completely simulate the propagation process of seismic waves in space under real conditions. The semi-auto coupling is wired, and it is cumbersome to use in a narrow space of the tunnel. The wireless detector is the trend of future engineering.

## Figures and Tables

**Table 1:** Specifications of the three-component piezoelectric sensor.

Description		Value
**Sensitivity (mV/g)**	X	2810
	Y	2830
	Z	2838
Full scale (g)		1.75
Frequency bandwidth (Hz)		10–5000
Resolution (g)		0.000006
Resonance frequency (Hz)		15 K

**Table 2:** The basic parameters of the TETSP-2 tunnel seismograph.

Number of Channels	Sample Interval	Dynamic Range	Working Time for One Charge
24	5.2 μs	126 dB	8 h
